# FICTION Technique—A Candidate for the Assessment of HER2 Status in Breast Invasive Carcinomas

**DOI:** 10.3390/medicina61061069

**Published:** 2025-06-11

**Authors:** Bogdan Fetica, Mihaiela Luminita Blaga, Adrian Pavel Trifa, Cosmina Maria Bocean, Ovidiu Balacescu, Annamaria Fulop, Bogdan Pop

**Affiliations:** 1 Department of Pathology, “Prof. Dr. Ion Chiricuta” Institute of Oncology, 400015 Cluj-Napoca, Romania; 2 Molecular Pathology Laboratory Pathos, 400394 Cluj-Napoca, Romania; 3 Medical Genetics Department 2—Morphological Microscopy, Faculty of Medicine “Victor Babeș”, University of Medicine and Pharmacy, 300041 Timișoara, Romania; 4 Medical Genetics Department, “Prof. Dr. Ion Chiricuta” Institute of Oncology, 400015 Cluj-Napoca, Romania; 5 Department of Functional Genomics, Proteomics and Experimental Pathology, “Prof. Dr. Ion Chiricuta” Institute of Oncology, 400015 Cluj-Napoca, Romania; 6 Pathological Anatomy Department 1—Morphological Sciences, Faculty of Medicine, “Iuliu Hațieganu” University of Medicine and Pharmacy, 400012 Cluj-Napoca, Romania

**Keywords:** in situ hybridization, fluorescence, breast neoplasms, immunohistochemistry, genes, erbB-2

## Abstract

*Background and Objectives*: The assessment of HER2 status in invasive breast carcinomas (IBCs) is critical for determining treatment strategies. The aim of this study was to evaluate the FICTION technique as a potential method for assessing HER2 status and to compare it with the standard sequential immunohistochemistry (IHC)–in situ hybridization (ISH) assays. *Materials and Methods*: This study included 49 patients diagnosed with invasive breast carcinomas. HER2 status was assessed using both IHC+FISH and FICTION techniques, and the results were compared. *Results*: Comparative analysis demonstrated an 83.67% categorical agreement between IHC and IF using the ASCO/CAP system. The percentage of cells showing any degree of HER2 protein expression was higher with IF (73.77%) than with IHC (60.71%) (*p* = 0.00026). The in situ hybridization assays showed an excellent agreement, with a 90% or higher concordance. The concordance of the ASCO/CAP group classification of cases using both ISH assays (FICTION and standard FISH) was high (85, 7%). Agreement was 100% for the final classification of cases (Her2 positive/negative). *Conclusions*: We compared standard tests for Her2 protein expression and the gene copy number with a modified FICTION protocol. The study showed moderate agreement between IHC and IF for Her2 protein and excellent agreement between FISH and FICTION ISH for the gene copy number. Final Her2 status was unaffected by low IF IHC concordance. Optimizing the FICTION protocol could improve results. Combining protein and gene assays may enhance IBC patient stratification.

## 1. Introduction

Breast cancer is the most commonly diagnosed cancer type and the main cancer-related cause of death in females. Hormone receptor expression and HER2 status remain the most important, validated predictive factors, and their determination is used to guide endocrine and targeted therapy. Accurate determination of HER2 status in these patients is crucial, as the advent of HER2-targeted therapy has significantly altered the prognosis for this subtype of breast cancer. Over time, several testing methods have been developed to identify protein expression, such as immunohistochemistry (IHC), or to detect gene copy number gains or amplification, which help in interpreting IHC equivocal results.

In situ hybridization (ISH) techniques, both brightfield and fluorescent, are now widely used to evaluate the HER2 copy number, particularly for predicting and assessing patient therapy outcomes. The American Society of Clinical Oncology/College of American Pathologists (ASCO/CAP) has issued several updates regarding the interpretation of IHC and ISH to standardize the evaluation process for Her2 in breast cancer [1,2,3,4].

The 2018 ASCO/CAP focused update of the HER2 testing guidelines emphasized the need for parallel assessment of IHC and in situ hybridization (ISH) results in invasive breast carcinoma (IBC) testing. The interpretation of ISH results must be performed for certain groups in accordance with IHC results [1].

The 2023 ASCO/CAP guideline update acknowledged and emphasized the clinical need for HER2 IHC-based assays to identify cases with scores of 1+ and 2+ (lacking HER2 gene amplification by in situ hybridization (ISH)) in the context of the results of the DESTINY-Breast04 trial. The updated guidelines underlined the fact that the results of the trial did not identify a new predictive biomarker threshold for the response to therapy-disrupting HER2 signaling pathways [2,3]. On the other hand, the inclusion criteria of the trial made the distinction between scores 0 and 1+ clinically relevant [2,3]. As the focus and the purpose of current IHC assays for Her2 detection are to select overexpression (3+) cases, there is a concern that using these assays for distinguishing between very low 0 and 1+ scores would yield suboptimal results [2]. Furthermore, a study aimed at assessing current IHC assays in the selection of patients with breast cancer eligible for trastuzumab deruxtecan therapy found a very low concordance for 0 and 1+ scores [4].

In this context, new methods for Her2 testing have been employed that are aimed at further subclassifying HER2 unamplified breast cancer cases [5]. This further stratification of cases is supported by the results of the DESTINY-Breast06 trial, which showed similar benefits for patients with Her2 1+, 2+ (unamplified cases) and ultra-low Her2 cases defined as 0+ (with membranous staining) [6,7].

In the context of the latest two major updates of the Her2 guidelines, which require that the final ISH results for groups 2–4 be based on a concurrent review of IHC, and the evolving clinical requirements for further Her2 testing stratification, we considered investigating a testing method that combines immunofluorescence assays and FISH (fluorescent in situ hybridization), called the FICTION technique (fluorescence immunophenotyping and interphase cytogenetics as a tool for the investigation of neoplasms). This technique allows simultaneous evaluation of protein expression and gene copy number assessment. Past uses of this technique have been mostly focused on tumor cell identification by means of immunofluorescence (IF) and gene copy number evaluation rather than the usage that we assessed in this study, the combined evaluation of Her2 protein expression and HER2 gene copy number, with one notable exception [8,9,10]. A literature review identified only one other study aimed at assessing Her2 status in IBC using FICTION [10].

The aim of this study was to evaluate the FICTION technique as a possible candidate for the assessment of HER2 status in invasive breast carcinomas (IBCs). To achieve this aim, we applied a modified FICTION protocol to IBC samples for the determination of HER2 status and compared the results to the standard sequential IHC and ISH testing method.

## 2. Materials and Methods

### 2.1. The Study Group

We selected 49 patients diagnosed with IBCs in the pathology department of our institution between January 2019 and October 2020. In this study, we included only cases that were evaluated by morphological examination and by using ancillary techniques, immunohistochemistry (IHC) and in situ hybridization (ISH), for the evaluation of estrogen receptor (ER) expression, progesterone receptor (PR) expression, Her2 protein expression and the KI-67 proliferation index, and for which the slides were available for reviewing.

All the tissue specimens sampled were fixed in 10% neutral buffered formalin for 6 to 72 h, followed by routine processing and paraffin embedding.

### 2.2. Routine Microscopic Examination of Invasive Breast Carcinomas (Standard IHC and ISH Ancillary Testing)

The histopathological examination was carried out according to the diagnostic protocol of the pathology department and followed the WHO 2019 recommendations [11].

Immunohistochemical (IHC) staining for ER, PR and KI-67 was performed in accordance with the laboratory’s IHC staining protocol used for routine clinical practice. IHC staining for HER2 testing was performed by an automatic IHC technique using the Ventana Benchmark Ultra staining machine and (4B5) rabbit monoclonal primary antibody. The Her2 automated IHC protocol employed heat-induced epitope retrieval (HIER) for CC1 cell conditioning (Ventana, Roche Diagnostics, Basel, Switzerland) for 36 min (maximum temperature 95 °C). Incubation with the RTU primary antibody was performed for 12 min at 36 °C. The visualization system used was UltraView Universal DAB Detection (Ventana): 8 min polymer and 8 min chromogen incubation at 36 °C with CuSO4 enhancement. Counterstaining was performed using Hematoxylin II solution (Ventana, Roche Diagnostics, Basel, Switzerland).

The ISH method used for HER2 gene copy number assessment was FISH, with the PathVysion HER-2 DNA Probe Kit (Abbott Molecular, Abbott Park, IL, USA).

The evaluation of estrogen receptors (ER), progesterone receptors (PR), KI-67 and HER2 receptors was performed according to the recommendations of ASCO/CAP [1,12]. Each Her2 IHC slide included an external control consisting of a tissue core with an IHC 3+ (positive) IBC.

### 2.3. The FICTION Staining Protocol

The FFPE specimens containing IBC samples were assessed using the FICTION technique. For this combined technique, we used a modified staining protocol, presented below:

1. From the paraffin blocks, sections were cut at 4 μm and spread on silane-coated slides,
2. After sectioning, the slides were incubated for at least 2 h at 56 °C or overnight at 37 °C for aging,
3. The slides were immersed in three consecutive baths of xylene, with 5 min for each bath,
4. The slides were then passed through two baths of absolute ethyl alcohol, with one minute in each bath,
5. The slides were washed in tap water,
6. The slides were placed in a steel holder, which was then placed in a Coplin jar containing citrate buffer solution, pH = 6, 0.01 M,
7. The jar was boiled using a pressure cooker at 100 °C for 20 min,
8. The pressure cooker was cooled under cold tap water and the steam gradually released,
9. The lid of the pressure cooker was opened,
10. The slides were moved to a Coplin jar with distilled water for 5 min,
11. The slides were then dried at room temperature for 10 min,
12. The slides were then immersed for 10 min in acetone,
13. Washing of the slides was performed in 100 mL of PN buffer [13],
14. Dehydration of the slides was performed in 70%, 85% and 96% ethanol, and the duration for each bath was 2 min at room temperature,
15. The slides were dried at room temperature for 5 min,
16. The slides were then hybridized by applying a 10 µL hybridization probe (PathVysion LSI HER-2/neu SpectrumOrange/CEP 17 SpectrumGreen Probes, Abbot molecular, Abbott Park, IL, USA),
17. The slides were next covered with a Ø 10 mm × 10 mm coating glass,
18. Sealing of the entire hybridization region was performed using Fixogum™ rubber cement,
19. The slides were transferred to a hybridization unit (Thermobrite, Abbot molecular, Abbott Park, IL, USA), where denaturation was performed for 7 min at 75 °C,
20. The hybridization process was performed for 16 h (overnight) at 37 °C in the hybridization unit,
21. The next day, two Coplin jars containing wash buffer (2X SSC/0.3% NP-40) were prepared; one was immersed in a water bath at 72 °C, and the other jar was left at room temperature,
22. Using tweezers, the Fixogum™ rubber cement and the glass coverslips were carefully removed; then, the excess probe was washed in the Coplin jar containing wash buffer solution at 72 °C for a duration of 2 min and 15 s, and the slides were then moved to the room temperature Coplin jar,
23. The slides were washed with 100 mL of PN buffer,
24. The slides were incubated with 100 µL of the primary antibody solution for immunofluorescence staining of the section and incubated for 30 min away from light (recombinant anti-ErbB2/HER2 antibody [clone CAL27] (ab237715), rabbit monoclonal, dilution 1:50, Abcam, Abcam Limited, Cambridge, UK),
25. The slides were then washed with 100 mL of PN buffer,
26. Incubation with 100 µL of the secondary antibody solution for 30 min (goat anti-rabbit IgG H&L (FITC) (ab6717), 1:10 dilution) followed,
27. The slides were washed in a Coplin jar with 2 × SSC for 10 min,
28. The slides were then completely air dried,
29. Nuclear counterstaining and mounting were performed using DAPI counterstain (4,6-diamidino-2-phenylindole) in phenylenediamine dihydrochloride, glycerol and buffer,
30. The slides were then coverslipped and sealed and stored for at least 30 min at 4 °C before the examination.

### 2.4. Preparation of the PN Buffer Solution [13]

PN buffer: 13.8 g of sodium acid phosphate (NaH_2_PO_4_ × H_2_O), 14.2 g of disodium hydrogen phosphate (Na_2_HPO_4_), adjust to 1000 mL of distilled water, pH 8.0, store at room temperature for 1 year [13].

### 2.5. Comparing Standard IHC + ISH Assays to the FICTION Technique

**Figure 1:**
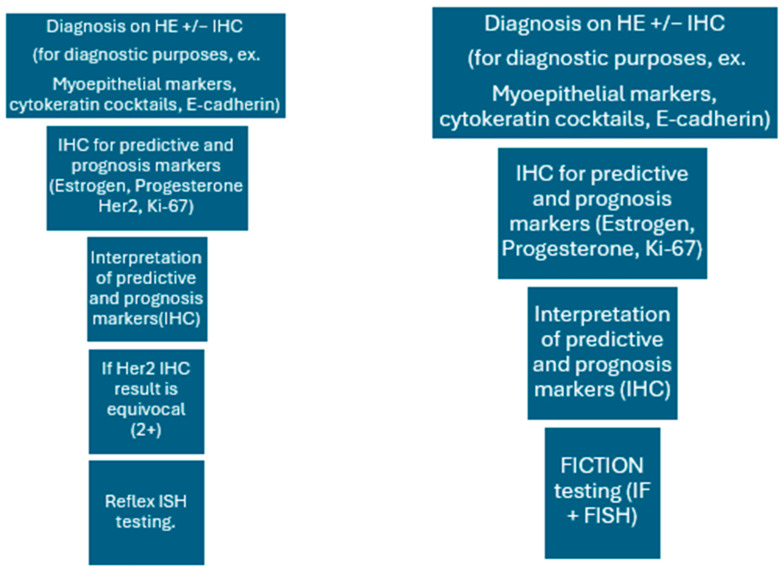
Comparative workflow for standard IHC + ISH evaluation and FICTION.

All cases underwent standard ancillary automated testing for the expression of Her2 protein using the 4b5 clone (Ventana). The slides were examined by senior pathologists from the pathology department in our institution and were scored and reported according to the ASCO/CAP guidelines [1]. The results were collected from the pathology department database. All cases were tested using FISH by employing the standard protocol used in our department. The IHC and FISH techniques were performed in a sequential manner, with IHC performed initially and another section used for the FISH technique (Figure 1). Prior to the examination of the FISH slides, the examiner reviewed each IHC slide and identified areas of interest with the highest protein expression. The formal counting of signals in the cells required for HER2 scoring was undertaken after a whole slide review of each FISH specimen to identify areas with the highest copy number and to coordinate the ISH slides with the IHC results. Reporting of the FISH results and the final concurrent IHC-FISH category was performed according to ASCO/CAP guidelines [1].

The FICTION technique was performed using a single section of the specimen according to the above-mentioned modified protocol. The examination was performed simultaneously for protein expression and copy number evaluation. Reporting of the immunofluorescence Her2 protein expression and HER2 copy number evaluation assessed using the FICTION technique also followed the ASCO/CAP guidelines [1]. Supplementary data collected was represented by the percentage of tumor cells showing any degree of protein expression, meaning that any tumor cell expression was considered and added to the total tally of tumor cells expressing Her2 protein. This was performed for the IHC-stained slides and for the assessment of the immunofluorescence (IF) protein expression observed on the FICTION-tested slides.

The results of the IHC and IF Her2 protein expression levels were assessed by comparing the percentage of cells expressing Her2 protein and by means of comparing the categorial assignment of cases using the ASCO/CAP four-tier system. Numerical data were compared using *t*-Test: Paired Two Sample for Means, Bland–Altman plots and Pearson’s correlation coefficient. Categorial agreement was assessed using Cohen’s kappa coefficient. Bland–Altman plots were generated using the digital supplementary content published by Andrew Carkeet [14]. In situ hybridization, both from standard FISH assays and FICTION assays, was compared in a similar way. The final categorization of cases according to ASCO/CAP 2018, using sequential standard IHC and ISH testing methods and FICTION, was performed, and the results were compared using Cohen’s kappa coefficient.

## 3. Results

### 3.1. The Study Group Histology Examination and Standard IHC and FISH Testing

The study group was represented by 49 patients diagnosed with breast cancer selected from the case files of the pathology department of the IOCN. The patients’ average age was 57.59, with a median of 60 years. Gender-wise distribution revealed a high female/male ratio (48:1).

Fifty-one IBC samples were selected for comparative analysis (one case was diagnosed with bilateral IBCs and one with bifocal IBC). Of these samples, 92% were represented by primary tumors, one sample was represented by lymph node metastasis and three samples were represented by skin fragments infiltrated by IBC. Most of the samples were represented by biopsy specimens (35/51 samples), the rest being excision specimens.

Tumor-type distribution showed that 74% of samples were classified as IBC of no special type/invasive ductal carcinoma (NST) (38/51), six cases were invasive lobular carcinomas (12%) and four cases were mixed invasive carcinomas (8%), represented by a primary NST component and a secondary histological type that exceeded 10% of the examined tumor volume area. The rest of the cases were represented by invasive micropapillary and cribriform carcinomas.

Most cases were classified as grade 2 Nottingham invasive carcinomas (57%, 29/51), followed by grade 3 cases (21%, 11/51) and nine cases as grade 1 (18%). In two cases, grading information was either unavailable or not provided.

Four out of the 51 samples were negative for estrogen receptors (7.84%) and nine out of the 51 samples were negative for progesterone receptors (17.64%). The Ki-67 values ranged from 5% to 65%, with a mean of 26% and a median of 21%.

Standard HER2 IHC expression was 2+ (equivocal) in 39/51 samples (76.47%), 10 cases were negative (1+, 19.6%) and 2 samples were positive by IHC (3+, 3.92%).

FISH analysis allowed the classification of cases in the ASCO/CAP 2018 groups. Group 1 represented 18% of samples (9 samples), Group 3 represented 12% of samples (6 samples), Group 4 represented 27% of samples (14 samples) and Group 5 represented 43% of samples (22 samples). The final classification of cases showed a HER2 positivity rate of 27.45% (14/51 samples).

### 3.2. Comparison of Standard IHC Her2 Testing Results and IF Results in the FICTION Assay

Comparative analysis was performed on 49 samples due to invasive carcinoma exhaustion or due to complete sample exhaustion from the paraffin block. When comparing the IHC and IF results for the immunohistochemistry/immunofluorescence HER2 category, we observed an 83.67% categorial agreement between IHC and IF. Eight cases had discordant results: six cases were classified as 1+ by IHC and as 2+ by IF, one case was classified as 2+ by IHC and 3+ by IF, and one case was classified as 3+ by IHC and as 2+ by IF. Cohen’s kappa coefficient was equal to 0.533 (*p* < 0.0001).

**Figure 2:**
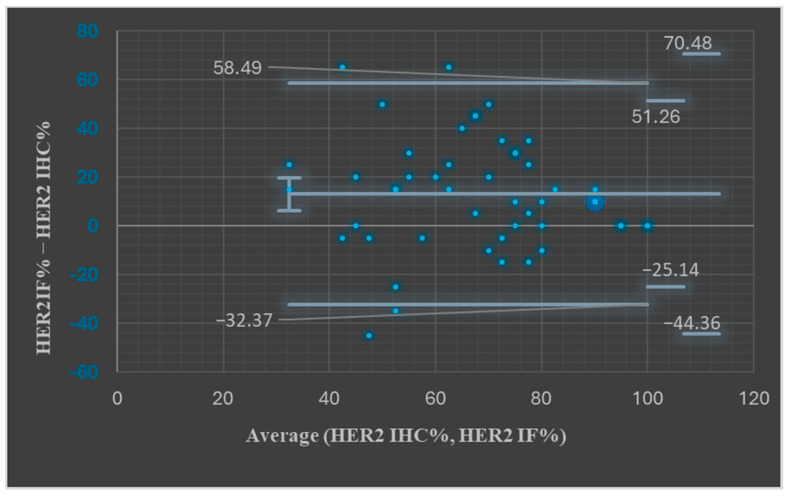
Bland–Altman plots assessing HER2 IHC% and HER2 IF% results.

We next assessed the percentage of tumor cells expressing the HER2 protein by IHC and by IF. Any degree of expression was considered. The mean expression for IHC was 60.71% and for IF was 73.77% (*t*-Test: Paired Two Sample for Means, *p* = 0.00026). Bland–Altman plots showed, for most paired measurements, that the values were between the upper and the lower LOA (45/49, 91.83%, Figure 2, *p* > 0.05).

### 3.3. Comparison of the Results of ISH Using the FISH Technique and ISH Results in the FICTION Assay

**Figure 3:**
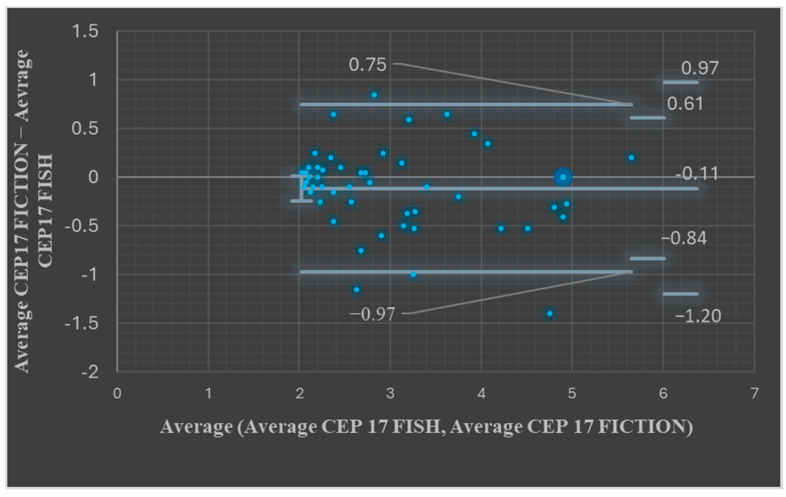
Bland–Altman plots comparing the FISH CEP17 average values and FICTION CEP 17 average values.

Comparative analysis of the ISH results in the FICTION and FISH assays showed an average number of signals for the centromeric probe of 2.95 signals/nucleus for the FICTION assay and an average of 3.06 signals/nucleus for the FISH assay. The median for the centromeric signals in FICTION was 2.7 and for the FISH assay was 2.75. The *t*-Test: Paired Two Sample for Means *p* value was 0.075, showing that the differences were not statistically significant. Bland–Altman plots showed, for most paired measurements, that the values were between the upper and the lower LOA (45/49, 91.83%, Figure 3, *p* > 0.05).

**Figure 4:**
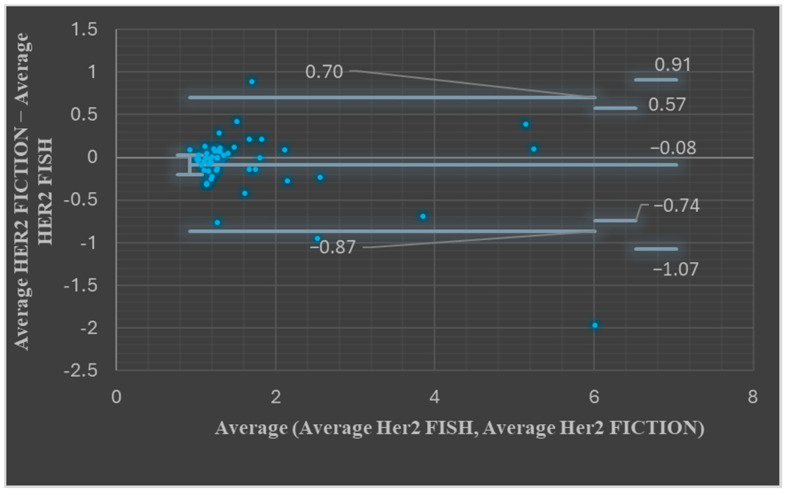
Bland–Altman plots assessing FISH HER2 average values and FICTION HER2 average values.

Next, we compared the HER2 copy number results in the FICTION and FISH assays. The average number of signals for the HER2 probe was 5.25 signals/nucleus for the FICTION assay and 5.05 signals/nucleus for the FISH assay. The median for the HER2 signals in FICTION was 3.85 and for the FISH assay was 4.05. The *t*-Test: Paired Two Sample for Means *p* value was 0.18, showing that the differences were not statistically significant. Bland–Altman plots showed, for most paired measurements, that the values were between the upper and the lower LOA (47/49, 95.91%, Figure 4, *p* > 0.05).

**Figure 5:**
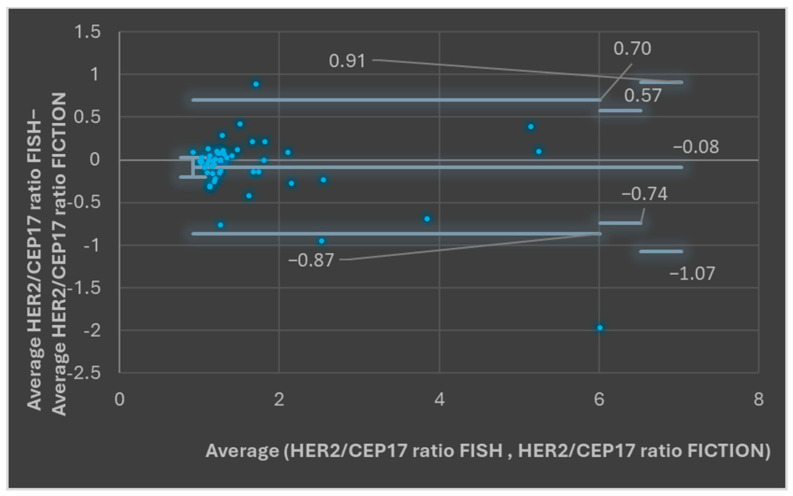
Bland–Altman plots assessing FISH HER2/CEP17 ratio average values and FICTION HER2/CEP17 ratio average values.

The combination of the two datasets (HER2 and CEP17 copy number), by assessing the HER2/CEP17 average values for FICTION and FISH assays, showed an average HER2/CEP17 ratio of 1.78 for the FISH assay and an average HER2/CEP17 ratio of 1.66 for the FICTION assay. The median for the HER2/CEP17 ratio in FICTION was 1.277 and for the FISH assay was 1.286. The *t*-Test: Paired Two Sample for Means *p* value was 0.148, showing that the differences were not statistically significant. Bland–Altman plots showed, for most paired measurements, that the values were between the upper and the lower LOA (46/49, 93.87%, Figure 5, *p* > 0.05).

**Figure 6:**
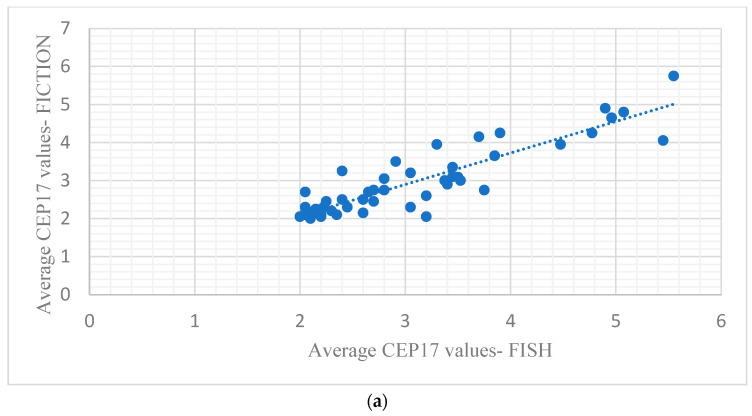

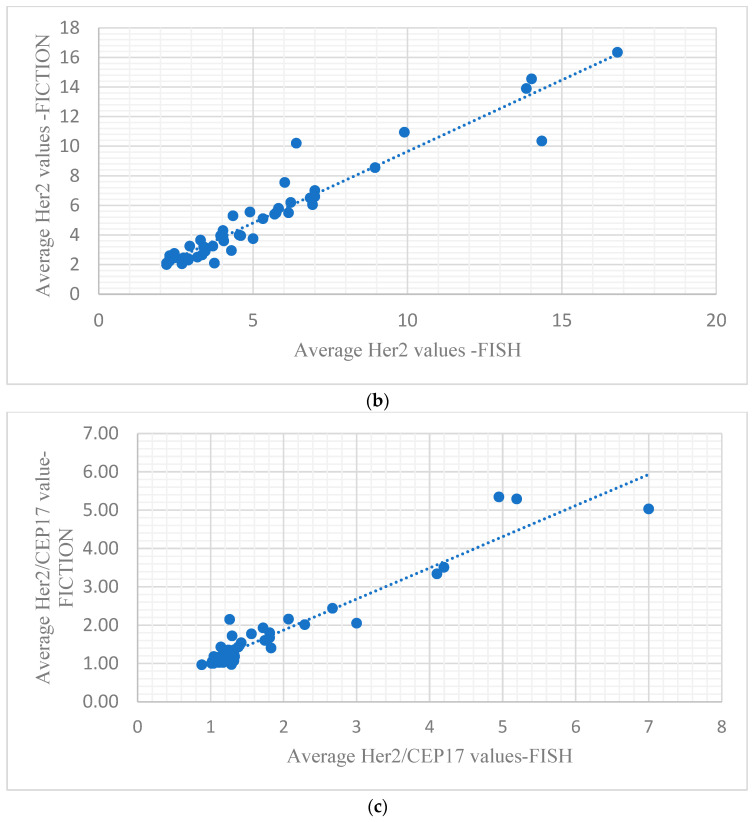
(**a**). Scatter plot of CEP17 average copy number by FISH and FICTION; (**b**) scatter plot of HER2 average copy number by FISH and FICTION; (**c**) scatter plot of HER2/CEP17 ratio by FISH and FICTION.

Pearson’s correlation coefficient for the average CEP 17 number of signals/cells was 0.90 (*p* < 0.001, Figure 6a), for the HER2 average copy number of signals/cells was 0.96 (*p* < 0.001, Figure 6b) and for the HER2/CEP 17 ratio was 0.95 (*p* < 0.001, Figure 6c).

### 3.4. Comparison of the Results of ASCO/CAP Groups Using the FISH Technique and FICTION Assay and of the Final Binary Classification (Interpretation) of HER2 Status

**Figure 7:**
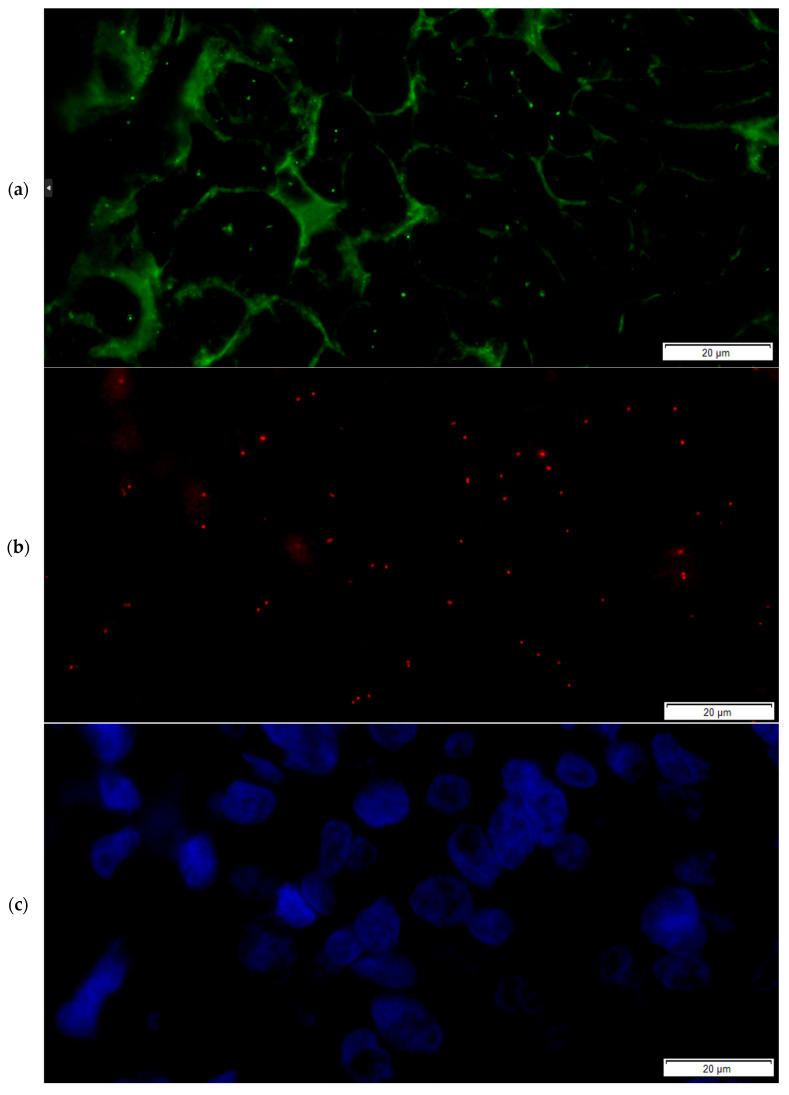

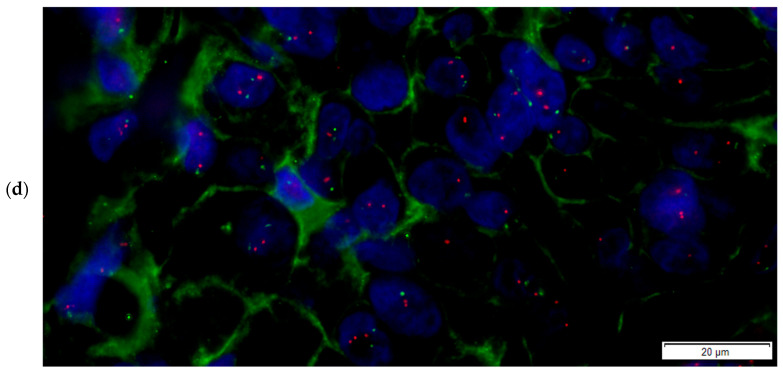
Representative pictures for FICTION-technique IF staining and ISH in invasive breast carcinomas: final categorical case result using FICTION was Group 5-Negative, with a 2+ IF score for Her2 protein expression. (**a**) Single-channel image, FITC filter, showing weak to moderate complete membranous green staining and small dot-like green signals corresponding to CEP 17; (**b**) single-channel image, TRITC filter, small dot-like red signals corresponding to HER2 gene; (**c**) single-channel image, DAPI, showing nuclear morphology for IBC; (**d**) combined multichannel image (FITC, TRITC and DAPI) showing Her2 1+ IHC and lack of Her2 gene amplification (ratio between red and green signals is below 2).

**Figure 8:**
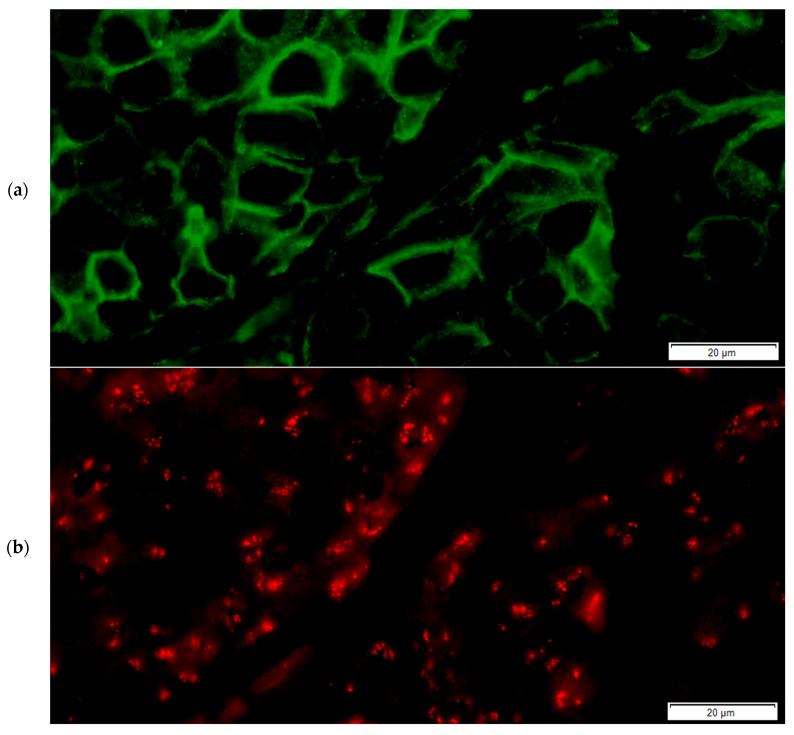

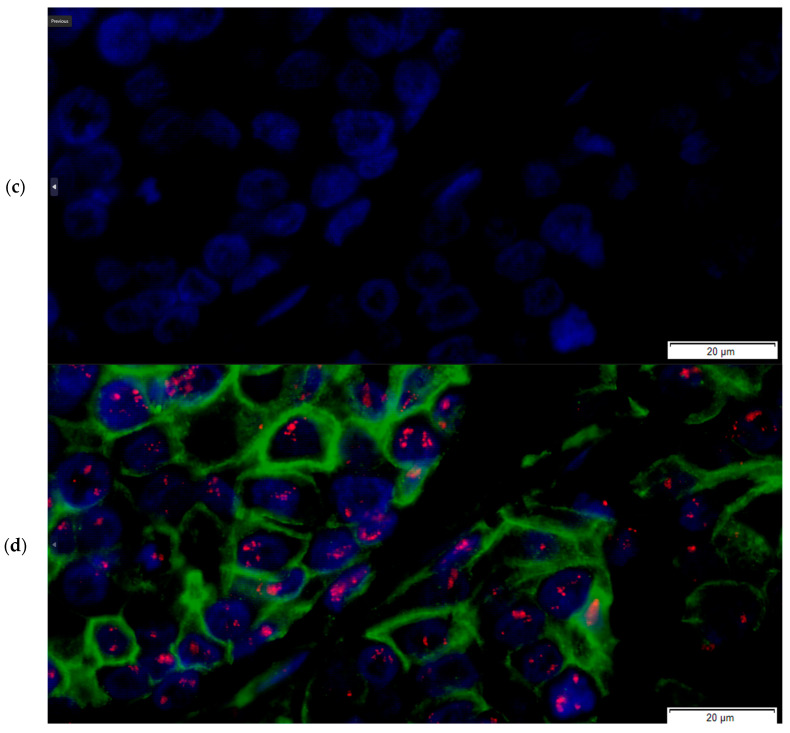
Representative pictures for FICTION-technique IF staining and ISH in invasive breast carcinomas: final categorical case result using FICTION was Group 1-Positive, with a 3+ IF score for Her2 protein expression. (**a**) Single-channel image, FITC filter, demonstrating strong complete membranous green staining and small dot-like green signals corresponding to CEP 17; (**b**) single-channel image, TRITC filter, showing multiple small dot-like red signals corresponding to HER2 gene forming clusters; (**c**) single-channel image, DAPI, showing nuclear morphology for IBC. (**d**) Combined multichannel image (FITC, TRITC and DAPI) showing Her2 3+ IF and Her2 gene amplification (two green signals and multiple red signals forming clusters, HER2/CEP 17 ratio above 2).

Based on the HER2/CEP17 ratio and the HER2 average copy number values, samples were then included in the ASCO/CAP ISH groups for both the FISH and FICTION assays (representative examples for the FICTION assay are presented in Figure 7 and Figure 8). When comparing the FISH and FICTION results, we observed a group agreement of 85.7%, with Cohen’s kappa coefficient equal to 0.785 (*p* < 0.0001). Five out of the six discordant cases were classified by FISH in Group 5 but in Group 4 by FICTION. One case was classified in Group 3 by FISH and in Group 1 by FICTION.

The final categorical inclusion of cases employing combined sequential IHC and FISH interpretation and FICTION interpretation was performed, and cases were classified as being eighter Her2 positive or Her2 negative. Analysis of the results showed a 100% agreement between the sequential IHC and FISH testing and FICTION and a kappa value of 1 (*p* < 0.0001). The detailed comparisons are summarized in Table 1.

## 4. Discussion

In the present study, we analyzed samples of IBC from 49 patients. Standard histopathological examinations and ancillary studies for predictive and prognostic markers were conducted using a usual sequence of IHC and FISH testing. The results of the standard ancillary tests for Her2 protein and the gene copy number were compared to the assessment performed using a modified FICTION protocol to assess the diagnostic yield of the method for Her2 testing in IBCs.

The comparative analysis for IF and IHC assays showed significant differences, with eight cases having discordant results. In most cases, IF overestimated HER2 protein expression compared to the IHC assay. This was also obvious when assessing the percentage of cells that showed any degree of HER2 protein expression using the two techniques, for which the differences were statistically significative (60.71% IHC vs. 73.77% for IF, *p* = 0.00026). Several factors can be taken into consideration when assessing these results. First, the primary antibodies used for the two assays were different (CAL 27 (Abcam) clone vs. 4B5 clone (Ventana)), and we have not yet been able to find data regarding expression in IBC using CAL27. Another factor with a potential influence on IF- IHC discrepancies is the fact that, when examining FFPE tissue in fluorescent light, there is also a high amount of background autofluorescence. We also did not employ a quenching solution in order not to alter the results of IF. Future investigations could try to employ these solutions to achieve lower background fluorescence. Also, in our current study, the secondary antibody used showed a variable degree of non-specific cytoplasmic staining, which should also be considered. Finally, and this is valid for all the rest of the comparative analysis that we performed, although the samples that were examined were taken from the same paraffin block, they were not identical, as tissue sectioning was performed at different moments. Nevertheless, the high number of cases with discordant results would most likely have an impact in routine clinical practice, especially in the context of identifying Her2-low and Her2-ultra-low cases. Although in our study there was no impact on the overall classification of the cases, Cohen’s coefficient (IF vs. IHC) seems also to point to a moderate agreement between the two assays in protein expression. It is worth noting that the FICTION technique allows the simultaneous evaluation of protein and gene copy number, so the real-life impact of these results will most likely not be related to an increased number of false-negative cases but to an overestimation of positive cases (an increase in false-positive cases).

Analysis of the CEP17 and HER2 average copy number and HER2/CEP 17 ratios showed that the differences observed were not statistically significant for ISH using standard FISH and FICTION, and the Bland–Altman plots showed that more than 90% of comparative assays fell within the upper and lower LOA, suggesting high concordance for the ISH assays. Comparison of the ASCO/CAP group classification of cases using both techniques showed a high agreement rate of 85.7%. Most cases misclassified by FICTION were Group 5 cases that were categorized as Group 4. One case was classified as Group 1 with FICTION and as Group 3 by FISH. This was more likely due to assessing different areas and sections from the same paraffin block, as explained earlier. However, the impact on the final classification of cases for HER2 status was not significant, as the agreement was 100% for the final classification of cases. This is an important finding in our opinion, as it argues in favor of the fact that the addition of an IF step to a standard FISH assay did not seem to have a major impact on the gene copy number in our study group.

The FICTION technique was first published by Weber-Matthiesen et al. [13], with the aim of allowing the simultaneous evaluation of morphologic, immunophenotypic and genetic features of individual cells. The protocol used in this study was based on the published work by Maciej Giefing and Reiner Siebert [9]. We made significant changes to the protocol in terms of antigen retrieval times and reagents and the IF and FISH step order (the current protocol performs ISH first and then IF), and we made major modifications to the post IF and FISH washing procedures. We were unable to conserve the membranous IF expression of HER2 protein by following the original protocol. Step-by-step analysis indicates that the high-temperature denaturation step at 75 °C is most likely the cause of IF membranous staining loss.

In this study, we set out to investigate the impact on the HER2 copy number and CEP 17 copy number of FISH assays when adding an IF stain to the protocol. Our main concern was represented by the fact that, in standard FISH assays, protease treatment usually digests most of the membrane and cytoplasm of the cells. For this reason, the published FICTION protocols do not employ protease treatments. However, there is an obvious need for a permeabilization step that allows the FISH probes to hybridize with the cell’s DNA. We showed that the membranous staining for HER2 is preserved after the permeabilization step, which employed the common procedure for antigen retrieval in IHC/IF.

Lottner. examined 215 invasive breast carcinomas using a tissue microarray and the FICTION technique and obtained a 97.7% concordance with the standard FISH examination, identifying all cases that showed overexpression and simultaneous gene amplification. Furthermore, the authors identified cases that showed gene amplification without protein overexpression. The protocol used by the authors was a FICTION protocol in which denaturation and hybridization were performed prior to IF antibody incubation [10]. This is the only other study that we were able to find that used FICTION to assess Her2 protein and the gene copy number. Our study revealed a higher impact on protein expression levels than in the data published by Lottner [10].

A similar technique involving double detection methods was used by Satosi Ikeda on invasive breast carcinomas, gastric carcinomas and Hodgkin lymphoma cases. The published protocol was based on performing IF in a chamber at 42 °C, followed by the FISH protocol. The correlation between gene amplification and IHC HER2 overexpression was performed by assessing 12 gastric and 20 breast biopsy specimens, and the results were compared to the results of standard laboratory assays. The comparison showed similar results with those of routine procedures. Furthermore, the potential of the IF and FISH combination was further exemplified by using a combination of cytokeratin antibody and a HER2 FISH probe on gastric carcinomas and a CD30 antibody and an IgH probe for lymphoma studies [15]. Gatta evaluated 20 breast samples using the FICTION technique and found a good agreement between the FISH and FICTION ISH results. The protocol used a cytokeratin antibody and a 10 min protease digestion time. The authors warned about extending the digestion times, as it would cause damage to the cytoskeleton and cause a false-negative IF result [8].

Alternatives to using fluorescent light examination have been proposed over the years [5,16,17,18,19,20,21]. Nitta. developed a protocol that allows simultaneous visualization of HER2 IHC and HER2 and chromosome 17 centromere using brightfield ISH, with results virtually equivalent to those of the single FDA-approved HER2 IHC and HER2 and CEN17 BISH assays. Furthermore, the staining protocol is fully automated [20]. Most of the validation studies have focused on comparing sequential IHC and FISH to determine Her2 gene and protein status and expression using synchronous gene novel detection methods, with good results. In all the above assays, IHC was performed prior to the ISH technique. To meet the need for patient stratification in Her2-low and ultra-low ranges, assays employing multiplex immunofluorescence and RNA-based approaches are being studied [22,23,24].

The FICTION technique, compared to current standard sequential IHC and ISH evaluation, has the potential to decrease the overall time required for the evaluation of Her2 status by decreasing overall protocol times and delays in requesting reflex ISH evaluation. Although the FICTION protocol is longer than the FISH protocol or the IHC protocol, it is significantly shorter than the time required to perform IHC and FISH separately. The FICTION technique also has the potential to be automated. In terms of costs, the technique should not be more expensive than IHC and ISH evaluated independently. Now, in most laboratories, the ISH method is used in IHC equivocal cases. Using FICTION on all cases requires a more in-depth analysis. The gains in turnaround times and with optimization in precision must be weighed against the costs. From a technical perspective, the technique should be accessible and straightforward to implement in any pathology laboratory that is familiar with IHC, IF and FISH techniques. Memon et al. published a review of the literature between 2001 and 2020 that noted that IHC^−^/ISH+ and IHC^+^/ISH^−^ discordances were seen in all antibody clones and ISH methods used in that period and suggested that routinely performing IHC an ISH could identify these incongruences [25].

Our study has several limitations. The number of cases included in the study was limited to 49 (51 samples), which had an impact on the comparison of our groups and on the ability of the study to formulate conclusions that can be generalized. The study used a whole slide analysis, limiting the number of samples that we were able to assess. We employed different primary antibodies (CAL 27 (Abcam) clone vs. 4B5 clone (Ventana)), as mentioned earlier, and we are not aware of any studies that compare the diagnostic yield of these two antibodies. The FICTION protocol is a manual protocol, while the IHC protocol using the 4B5 Ventana clone is a fully automated protocol. One of the caveats of not using enzyme digestion (protease) was the presence of higher levels of autofluorescence. We refrained from using quenching solutions because of the lack of information regarding their interaction with IF staining.

## 5. Conclusions

We compared standard ancillary tests for Her2 protein expression and the gene copy number with a modified FICTION protocol. Our study showed moderate agreement between IHC and IF results for Her2 protein expression and excellent agreement between standard FISH and FICTION ISH results, with minimal impact on the copy number of centromeric probes and HER2 probes. Final Her2 status was unaffected by the low IHC-IF concordance. Despite some limitations, optimizing and automating our FICTION protocol could improve results. Combining protein and gene assays should be explored for better IBC patient stratification.

## Figures and Tables

**Table 1:** Summary of the comparison between sequential IHC + FISH and FICTION.

	IHC +FISH	FICTION	*p* Value
Her2 protein expression(categorial)	83.67% agreement		<0.0001
	Cohen kappa 0.533		
Her2 protein expression(percentage of tumor cells)	60.71% mean expression	73.77% mean expression	=0.00026
	91.83% between upper and lower LOA (45/49 samples)		>0.05
CEP17—ISH	2.95 average CEP17 signals	3.06 average CEP 17 signals	>0.05
	91.83% between upper and lower LOA (45/49 samples)		>0.05
	Pearson correlation coefficient = 0.90		<0.001
HER2—ISH	5.05 average HER2 signals	5.25 average HER2 signals	>0.05
	95.91% between upper and lower LOA (47/49)		>0.05
	Pearson correlation coefficient = 0.96		<0.001
HER2: CEP 17—ISH	1.78 average HER2/CEP 17 ratio	1.66 average HER2/CEP 17 ratio	>0.05
	93.87% between upper and lower LOA (47/49 samples)		>0.05
	Pearson correlation coefficient = 0.95		<0.001
ASCO/CAP ISH groups	85.7% group agreement		
	Cohen’s kappa coefficient = 0.785		<0.0001
Final category (Her2 positive/Her2 negative)	100% agreement Cohen’s kappa coefficient = 1		<0.0001

## Data Availability

The raw data associated with this study can be obtained upon reasonable request addressed to the corresponding author.

## Abbreviations

The following abbreviations are used in this manuscript:

LOA	Limit(s) of agreement
IBC	Invasive breast carcinoma
CEP17	Centromeric enumeration probe for chromosome 17
FISH	Fluorescent in situ hybridization
FICTION	Fluorescence immunophenotyping and interphase cytogenetics as a tool for the investigation of neoplasms
IHC	Immunohistochemistry
ISH	In situ hybridization
FITC	Fluorescein isothiocyanate
TRITC	Tetramethylrhodamine
DAPI	4′,6-diamidino-2-phenylindole
